# Supplementary material for: 9p21 Locus Polymorphism Is A Strong Predictor of Metabolic Syndrome and Cardiometabolic Risk Phenotypes Regardless of Coronary Heart Disease
Source: Genes (Basel). 2022 Nov 27;13(12):2226. doi: 10.3390/genes13122226 (PMC9778176; doi:10.3390/genes13122226)
Supplement: Supplementary file 1 [file genes-13-02226-s001.zip › genes-2043810-supplementary.pdf]

genes

Supplementary Material

9p21 Locus Polymorphism is A Strong Predictor of Metabolic Syndrome and Cardiometabolic Risk Phenotypes Regardless of Coronary Heart Disease

Muhammad Mobeen Zafar [1], Muhammad Saqlain [2], Asad Mehmood Raja [1], Pakeeza Arzoo Shaiq [1], Muhammad Javaid Asad [1], Muhammad Kausar Nawaz Shah [3], Farah Fatima [4], Hadi Valadi [5] Muhammad Nawaz [5,*], Ghazala Kaukab Raja [1,*]

[1] University Institute of Biochemistry & Biotechnology, PMAS- Arid Agriculture University Rawalpindi, 46300, Pakistan
[2] Department of Biochemistry and Molecular Biology, University of Sialkot, Sialkot, 51040, Pakistan
[3] Department of Plant Breeding and Genetics, PMAS- Arid Agriculture University Rawalpindi, 46300, Pakistan
[4] Department of Pathology, Ribeirao Preto Medical School, University of Sao Paulo, Ribeirao Preto, 3900, Brazil
[5] Department of Rheumatology and Inflammation Research, Institute of Medicine, Sahlgrenska Academy, University of Gothenburg, Gothenburg, 41346, Sweden

* Correspondence: Muhammad Nawaz (muhammad.nawaz@gu.se), Ghazala Kaukab Raja (ghazala@uaar.edu.pk)

Citation: Mobeen Zafar, M.; Saqlain, M.; Mehmood Raja, A.; Arzoo Shaiq, P.; Javaid Asad, M.; Nawaz Shah, M.K.; Fatima, F.; Valadi, H.; Nawaz, M.; Raja, G.K. 9p21 Locus Polymorphism Is A Strong Predictor of Metabolic Syn-drome and Cardiometabolic Risk Phenotypes Regardless of Coronary Heart Disease. *Genes* **2022**, *13*, 2226. https://doi.org/10.3390/genes13122226

Academic Editor: Zsolt Ronai

Received: 3 November 2022
Accepted: 23 November 2022
Published: 27 November 2022

Publisher's Note: MDPI stays neutral with regard to jurisdictional claims in published maps and institutional affiliations.

Copyright: © 2022 by the authors. Submitted for possible open access publication under the terms and conditions of the Creative Commons Attribution (CC BY) license (https://creativecommons.org/licenses/by/4.0/).

Supplementary table for Figures 3 and 4

Table S1: Comparison of study variables in rs1333049 SNP minor/risk (C) allele and major (G) allele carriers and SD change from population means in C allele carriers

Study Variables	Non-Carrier (GG)	C Allele Carrier (GC + CC)	SD change from population means in C allele Carrier	*P*-value
Age (Years)	40.8	46.3	2.3	1.36E-04
Weight (Kg)	70.5	72.3	0.1	3.38E-04
SBP (mm/Hg)	126.2	128.4	-8.4	2.57E-02
DBP (mm/Hg)	82.1	84.6	2.2	1.10E-04
FBG (mg/dl)	95.9	102.4	2.1	3.48E-04
TG (mg/dl)	157	174.3	2.2	3.82E-02
TC (mg/dl)	193.9	215.6	-2.6	2.00E-04
HDL (mg/dl)	48.2	43.9	-1.7	4.55E-03
LDL (mg/dl)	112.1	126.4	1	4.70E-05

SBP; systolic blood pressure, DBP; diastolic blood pressure, FBG; fasting blood glucose, TG; triglycerides, TC; total cholesterol, HDL; high-density lipoprotein, LDL; low density lipoprotein.

Supplementary table for Figures 5 and 6

Table S2: Comparison of adiposity traits in rs1333049 SNP minor/risk (C) allele and major (G) allele carriers and SD change from population means in C allele carriers

Adiposity Indices	Non-Carrier (GG)	C Allele Carrier (GC + CC)	SD change from population means in C allele Carrier	***P-value***
BMI (Kg/m^2)	26.2	28.9	0.4	1.10E-04
WC (cm)	86.4	89.4	3.9	3.48E-04
WHR	0.82	0.84	-0.009	4.99E-04
VAI	2.1	2.4	0.1	4.00E-02
BAI	25.3	26.4	1.4	3.30E-02

BMI; body mass index, WC; waist circumference, WHR; waist to hip ratio, VAI; visceral adiposity index, BAI; body adiposity indices,
